# Vitamin D intoxication in patients with cystic fibrosis: report of a single-center cohort

**DOI:** 10.1038/s41598-021-87099-w

**Published:** 2021-04-08

**Authors:** Thomas Planté-Bordeneuve, Silvia Berardis, Pierre Bastin, Damien Gruson, Laurence Henri, Sophie Gohy

**Affiliations:** 1 Department of Pneumology, Cliniques universitaires Saint-Luc, Avenue Hippocrate 10, 1200 Brussels, Belgium; 2 Department of Paediatric Pneumology, Cliniques universitaires Saint-Luc, Brussels, Belgium; 3 Department of Clinical Biology, Cliniques universitaires Saint-Luc, Brussels, Belgium; 4 Department of Pharmacy, Cliniques universitaires Saint-Luc, Brussels, Belgium; 5 Cystic Fibrosis Reference Center, Cliniques universitaires Saint-Luc, Brussels, Belgium

**Keywords:** Endocrine system and metabolic diseases, Cystic fibrosis

## Abstract

Vitamin D toxicity is associated with accidental overdoses due to manufacturing or intake errors and its secondary hypercalcemia can result in severe morbidity. Although patients with cystic fibrosis are potentially at increased risk for this intoxication as prescription of vitamin D preparations is a common practice in this population, the frequency of such events is currently unknown. We performed a retrospective analysis of all the files of cystic fibrosis patients followed at the Cliniques universitaires Saint-Luc over a 10-year period, recording 25(OH)- and 1,25(OH)_2_vitamin D levels as well as demographic data, lung function tests, *Pseudomonas aeruginosa* infection and results from pharmacological analysis of magistral liposoluble vitamins preparations. A total of 244 patients were included in the study. 13 patients (5%) had serum vitamin D levels corresponding to vitamin D overdose. Patients who had experienced an overdose were more likely to be F508del homozygous or suffer from exocrine pancreatic insufficiency. 2 patients developed significant hypercalcemia necessitating monitoring and hospitalization. Errors in the preparation of magistral liposoluble vitamin pills were identified in several intoxicated patients. Retrospective assessment of the dosing errors with the local pharmacists showed that trituration and dosing errors were their most frequent causes.

## Introduction

Vitamin D (VD) toxicity requires serum levels of 25-hydroxyvitamin D (25(OH)D) above 150 ng/mL^1^ and is mainly associated with exposure to extreme doses due to manufacturing or intake errors^2^. Symptoms are related to secondary hypercalcemia caused by increased calcium intestinal absorption and release from bone stores^3,4^. VD supplementation is common in the cystic fibrosis (CF) population due to the high prevalence of vitamin D deficiency secondary to exocrine pancreatic insufficiency^5^, reduced fat stores and decreased sunlight exposure^6^. As a result of exocrine pancreatic insufficiency and secondary fat malabsorption, this supplementation is frequently combined with pancreatic enzymes and other liposoluble vitamins (A, E, K), either under the form of commercial products or, in countries where these are not available, under the form of compounding or magistral prescriptions. The latter is dependent on human intervention and has been associated with intoxications due to dosage errors^7^. Currently, real-life data regarding VD toxicity in CF is lacking. To investigate the prevalence of vitamin D intoxication, we performed a retrospective single-center analysis of all the CF patients followed at our tertiary care center over a period of ten years. Our study revealed an elevated number of cases of vitamin D overdosing and toxicity in this at-risk population secondary to dosing errors in magistral preparations of liposoluble vitamins.

## Methods

### Population

All files from patients who were followed at the Cliniques universitaires Saint-Luc’s Cystic Fibrosis Reference Center between 01/01/2009 and 31/07/2019 were retrospectively reviewed after obtaining the agreement from our local ethics committee (Comité d’Ethique Hospitalo-Facultaire Cliniques universitaires Saint-Luc n°2019/09JAN/003). All research was performed in accordance with the relevant guidelines and regulations. Due to the nature of the study, the need for an informed consent was waived by the ethics committee (Comité d’Ethique Hospitalo-Facultaire Cliniques universitaires Saint-Luc n°2019/09JAN/003). Patients were eligible for inclusion if they met the following criteria: diagnosis of CF confirmed by sweat and/or genetic testing and at least one measure of serum 25(OH)D. Demographic data (age, sex, duration of follow-up, genotype status, height/weight/BMI z-scores), paediatric status (defined as age below 16 years), pancreatic and nutritional status (presence of exocrine and endocrine pancreatic insufficiency, liposoluble vitamin supplementation) were available for all patients (n = 244), while respiratory parameters (forced vital capacity (FVC), forced expiratory volume in 1 s (FEV_1_)) were recorded in 214 patients (unavailable in 20 patients because of young age, two patients with mental retardation, one missing data during VD toxicity episode, one patient with unreliable measurements and six patients who had undergone lung transplantation). Infectious status (chronic *Pseudomonas aeruginosa* colonization) was collected for the non-transplanted patients (n = 238). In the overdosed group, serum creatinine and calcium corrected for albumin were available for all patients except one during the episode. Data at the time of overdosing was compared with the latest available data of patients without pathological VD levels.

### Vitamin D measurements

Vitamin D measurements were performed during yearly screening, as recommended in this population^6^ (n = 242) or during the work-up for hypercalcemia (n = 2). Between January 2009 and June 2014, 25(OH)D measurements were performed by the LIAISON total VD assay (DiaSorin, Stillwater, MN, USA). After June 2014, 25(OH)D was measured using an electro-chemiluminescent assay on the Cobas e602 (Roche, Switzerland). Very high levels of 25(OH)D were confirmed through a vitamin D standardization program with traceable liquid chromatography and tandem mass spectrometry. Before January 2015, levels of 1,25-dihydroxyvitamin D (1,25(OH)_2_D) were measured thanks to the Diasorin radioimmunoassay (DiaSorin, Stillwater, MN, USA). After this date, 1,25(OH) concentrations were determined through a LIAISON XL 1,25(OH)_2_D assay. VD intoxication was defined as 25(OH)D levels superior to 140 ng/mL, which was the saturation limit of our test. For ten patients exceeding this threshold, exact 25(OH)D measurements were available while for three no exact dosage was performed after the saturation point.

In five patients, further analysis of the medication was performed by dosage of cholecalciferol in the pills. Our pharmacology department retrieved patients’ medication when VD toxicity was diagnosed and sent them to the Belgian association of pharmacology laboratory where further composition determination was performed using liquid chromatography with UV detection.

### Statistics

Data are presented as median with interquartile range (IQR). All statistical analysis was performed using Graphpad Prism version 8.0.2 (GraphPad Software, Lajolla, California, U.S.A.). Mann–Whitney U-test was used for two groups comparison when data was continuous. For categorical data, a Chi-squared or a Fisher’s exact test when applicable was chosen. A p-value < 0.05 was considered significant.

## Results

Our analysis identified 244 eligible patients who presented themselves at our clinic at least once over the 10 years period and had at the minimum one 25(OH)D concentration measured. 13 patients (5.3%) met the criteria for severe vitamin D overdosing (i.e. 25(OH)D > 140 ng/mL) with 11 dosages performed during routine vitamin D measurements and 2 during a work-up for hypercalcemia. There was no difference between patients under and above this threshold regarding age, sex, weight, height, or BMI z-score or concerning respiratory parameters such as FEV_1_, FVC and chronic *Pseudomonas aeruginosa* colonization. Furthermore, no difference in the number of patients suffering from advanced CF lung disease, as recently defined by the Cystic Fibrosis Foundation^8^, could be observed. Patients with excessive 25(OH)D measurements were more likely to be F508del homozygous, to have exocrine pancreatic insufficiency and to be treated with combined liposoluble multivitamin (A, D, E, K) preparations (Table 1). Notably, none of the patients were taking VD-fortified enteral caloric supplementation, enteral solution or milk at the time of intoxication.

**Table 1:** Characteristics of the patients with (n = 13) and without (n = 231) VD intoxication.

	No VD intoxication (n = 231)	VD intoxication (n = 13)	p-value
Age, years Median (IQR)	23 (13–34)	20 (9–33)	0.41
M/F, n	115/116	6/7	0.99
Paediatric status, n (%)	74 (32.03)	5 (38.46)	0.76
F508del/F508del, n (%)	92 (39.83)	10 (76.92)	0.01
ppFEV1, % Median (IQR)	86 (63–104) (n = 205)	81 (73–94) (n = 9)	0.77
ppFVC, % Median (IQR)	94 (82–107) (n = 205)	112 (87–114) (n = 9)	0.10
ACFLD, n (%)	25 (12.20) (n = 205)	0 (0) (n = 9)	0.60
Weight, z-score Median (IQR)	− 0.48 (− 1.25–0.36)	− 0.30 (− 1.35–0.05)	0.99
Height, z-score Median (IQR)	− 0.64 (− 1.30–0.16)	− 0.10 (− 1.35–0.20)	0.61
BMI, z-score Median (IQR)	− 0.2 (− 1.00–0.57)	− 0.10 (− 0.85–0.10)	0.62
Insulin use, n (%)	39 (16.88)	2 (15.38)	0.99
*Pseudomonas* chronic infection, n (%)	54 (23.89) (n = 226)	5 (41.67) (n = 12)	0.17
Pancreatic insufficiency, n (%)	176 (76.19)	13 (100)	0.04
Combined liposoluble vitamins supplementation, n (%)	172 (74.46)	13 (100)	0.04
Isolated VD supplementation, n (%)	42 (18.18)	0 (0)	0.13
VD dose, IU/d Median (IQR)	4800 (3000–8000) (n = 213)	4000 (2500–6500)	0.43

n is specified if data are missing.

*VD* vitamin D, *ppFEV*_*1*_ percent predicted values of forced expiratory volume in 1 s, *ppFVC* percent predicted values of forced vital capacity, *ACFLD* advanced CF lung disease, *M* male, *F* female, *IQR* interquartile range, *IU/d* international unit/day.

The median serum 25(OH)D level in the overdosed patients group for which an exact dosage was available (n = 10) was 185.5 ng/mL (IQR 150–316). While all patients in the latter group received vitamin D supplements, 213 patients in the group without intoxication received this medication. The prescribed doses of vitamin D supplements were then compared between the two groups (last known dose for the group < 140 ng/mL versus prescribed dose at the time of overdosing for the group > 140 ng/mL) and no difference could be found when considering the whole unintoxicated group (p = 0.80) or the subgroup of patients to whom D_3_ supplementation was prescribed (p = 0.43). 1,25(OH)_2_D levels were available for seven patients at the time of overdosing and were mildly elevated (median fold change compared to the Upper Limit of Normal (ULN) 1.78 (1.13–3.64)). Serum concentrations of vitamin A and E were determined in 11 of the 13 patients at the time of intoxication, revealing a ratio superior to 1.5 times the last measurement before the overdosing in only one patient (1.96 and 4.15 times respectively). In overexposed patients, serum creatinine was within normal values while median serum calcium was at the upper limit of normal (Table 2).

**Table 2:** Description of the profile of vitamin D intoxicated patients.

	Value
VD dose prescribed, IU/d Median (IQR)	4000 (2500–6500)
Measured VD dose per pill, IU/d Median (IQR)	157,358 (100,279–184,774) (n = 5)
VD intake per day, IU/d Median (IQR)	196,703 (128,295–389,358) (n = 5)
Vitamin A or E > 1.5 last dosage, n	1 (n = 11)
Serum 25(OH)_1_VD, ng/mL Median (IQR)	185.5 (150–316)
Serum 1,25(OH)_2_VD, Fold change to ULN Median (IQR)	1.78 (1.13–3.64) (n = 7)
Serum calcium corrected for albumin, fold change to ULN Median (IQR, n)	1.00 (0.95–1.10) (n = 12)
Serum creatinine, mg/dL Median (IQR, n)	0,87 (0.47–1.12) (n = 12)
Symptoms, n	2
Hospitalization, n	2
Serum 25(OH)_1_VD > 100 ng/mL after 4 months	4

The median D_3_ intake per day was calculated by multiplying the median VD dose per pill by the number of pills taken per day.

n is specified if data are missing.

*VD* vitamin D, *IQR* interquartile range, *IU/d* international unit/day.

Only two patients developed clinical toxicity (15.3%) with symptoms under the form of polydipsia (n = 2), vomiting (n = 1) and polyuria (n = 1). Both patients presented with severe hypercalcemia (corrected serum calcium 3.83 and 3.98 mmol/L respectively), hypercalciuria and acute kidney injury AKIN stage 2 and 3. They also displayed electrocardiographic changes with shortened QTc (n = 2), ST-segment elevation (n = 1) and biphasic T-waves (n = 1). VD supplementation was discontinued, the two subjects were hospitalized for correction of their calcium and received saline perfusion associated with diuretics, bisphosphonates, and corticosteroids as well as calcitonin for one. Calcium rapidly returned to normal values (duration of hypercalcemia respectively 1 and 3 days) as did the other clinical abnormalities. Over the following months, no rebound hypercalcemia was noted. In our affected population, after the discovery of elevated VD levels, VD supplements were either discontinued (n = 9) or drastically diminished (n = 4) with normalisation of the 25(OH)D levels in the following months. Repeat values for VD were available at 4 and 8 months for 10 out of 13 patients, revealing that only four patients had levels above 100 ng/mL after 4 months while none exceeded this value after 8 months. Finally, as nephrolithiasis^9^ and nephrocalcinosis^10^ are potential complications of VD intoxication, the yearly abdominal ultrasonography of the patients were assessed at the time of intoxication (median time-lapse from intoxication 0 days (0–56)) and at least 6 months after the event. Kidneys were evaluated in 10 out of 13 patients and none displayed compatible signs.

The main cause of these overdoses was manufacturing errors of the magistral liposoluble vitamins preparations (eight confirmed cases). The median occurrence rate per year was 2 and events ranged from 2013 to 2019. No spatial or temporal clustering could be found. Analysis of the VD dose contained in the pills was performed in 5 patients and revealed extremely high quantities of VD (median 157,358 IU (100,279–184,774 IU)). In the remaining patients, the local preparing pharmacist reported a ten times dosage error for two of them, corresponding to a potential 40,000 IU per pill, the medical record of one patient reported a 54-fold increased concentration in the medication (no exact number could retrospectively be found) clinical suspicion of overdosing was high in one subject and no aetiology could retrospectively be found in 4. Contact was taken by our pharmacy department with every local pharmacist and potential errors were reviewed with them. Trituration (n = 3) and various dosage errors (n = 3) were two commonly identified mistakes.

## Discussion

This retrospective single-center study showed an unexpected high prevalence of VD-overdosing in patients with CF, affecting more than 5% of our cohort over a 10-year period. Clinical toxicity was only witnessed in two patients but was associated with severe hypercalcemia, resulting in acute kidney injury and ECG-changes, requiring hospitalization. The main cause of these overdoses were accidental errors in the magistral preparations of liposoluble vitamins supplementations by local pharmacists.

Vitamin D_3_ (D_3_) is currently the recommended form of substitution for patients with CF^6^ and it can be obtained from the transformation of 7-dehydrocholesterol to D_3_ by UV-light in the skin or through dietary intake. Circulating D_3_ undergoes a first hydroxylation step in the liver by vitamin D 25-hydroxylase, yielding 25(OH)D before being hydroxylated in the kidney to 1,25(OH)_2_D, its effective form under normal conditions^3^. This metabolite is then capable of binding the vitamin D receptor (VDR) with a strong affinity, influencing the behaviour of cells implicated in bone turn-over, calcium-phosphate metabolism or immunity^9^. The first hydroxylation step is much less controlled than the second one, resulting in high 25(OH)D levels in case of D_3_ overdose^3^ while 1,25(OH)_2_D levels remain normal or are slightly elevated^11^. This excessive 25(OH)D is subsequently able to exceed the binding capacity of its transporter protein, the vitamin D-binding protein (VDBP) and to interact with the VDR, for which it has some affinity^11^. Inappropriate interaction with the VDR can then mediate pathologic intestinal calcium reabsorption and mobilization from bone^3^, resulting in hypercalcemia and gastro-intestinal (nausea, vomiting, constipation, polydipsia), neurological (confusion, depression, hallucination, coma), cardiac (QT shortening, ST-segment elevation, bradyarrythmias), or renal (polyuria, nephrocalcinosis, acute kidney injury) symptoms.

Although VD supplementation is common practice in the CF population, reports on toxicity are scarce^12^ even in patients receiving elevated doses^13,14^ and, similarly to the general population, toxic levels should result from the prolonged use of inadequately dosed supplements^2,11^. Additionally, despite the presence of severely elevated 25(OH)D levels, clinical complications remain a rare event. In the general population, the estimated frequency of elevated 25(OH)D and hypercalcemia ranges from 0.2 to 3.9% of patients with elevated serum 25(OH)D concentrations^15,16^ while symptoms are only present in 0.05–0.5%^15,17^. There are currently no real-life reports available to estimate if patients with CF are at particular risk for VD toxicity complications. Our series shows that two out of 13 patients with 25(OH)D > 140 ng/mL developed clinically significant hypercalcemia. Although this high percentage probably overestimates the frequency of this complication due to the small overall sample size, CF-specific factors (low body fat percentage, facilitated dehydration) could theoretically influence disease course by favoring firstly high serum VD levels and secondly kidney insufficiency. Importantly, these results should not deter physicians to pursue adequate VD supplementation, given (a) the potential benefits of sufficient VD levels, (b) the general safety of 25(OH)D intake and (c) the identification of compounding mistakes as the main contributor to the overdoses in our study.

Specific data is currently lacking regarding the causes of VD overdosing in patients with CF but reports concerning the general population suggest manufacturing errors of supplements as one of the main contributors^2,18^. Although considered a safe alternative, magistral preparations carry with them the inherent risk of human error and have been punctually linked to diverse accidental intoxications^19,20^. The CF-population requires specific medications and formulations for which hand-made preparations are an alternative when no reimbursed commercial alternative is available and are thus potentially more exposed to this risk.

In our population, the median prescribed dose of 4000UI of D_3_ could not explain the observed 25(OH)D levels, and further analysis of the available vitamin D concentrations contained in the compounded prescribed pills revealed that patients were taking a median 69 times the prescribed dosage. These errors concerned predominantly vitamin D and not the other prescribed liposoluble vitamins as no important elevation in the serum concentrations for vitamin A or E could be found in 12 of the 13 patients. While the exact cause of this predominance is unclear, as preparations for liposoluble vitamins require very similar steps, our pharmacy department was able to identify measurements and trituration errors as the cause of these intoxications. Given the isolated nature of D_3_ intoxication (as other vitamins were largely unaffected), vitamin D could be withdrawn from the magistral preparation and prescribed under a commercially available form, as these would be less prone to dosing errors.

Supplementation with high vitamin D preparations have been associated with adverse outcomes in a geriatric population^21^. When we analyzed the current characteristics of our overdosed patients, we could not find any differences with the rest of our population regarding weight, height or BMI z-score, chronic *Pseudomonas* infection, FEV_1_, FVC, or the proportion of advanced CF. The effect of very high VD levels on bone mineralization are a theoretical concern as VD-associated hypercalcemia results from the mobilization of bone stores next to intestinal absorption^3^. Given the accidental nature of VD overdosing, data regarding this potential effect is scarce but patients treated with high dose VD_3_ supplements (mean VD level: 57 ng/mL) showed limited effects on DXA-measured bone mineral density (BMD), and a decrease in volumetric BMD^22^. In our study, BMD measures before and after the intoxication were only available in two patients, showing diverging results with, in one case, a stability (lumbar spine + 0.4%, hip + 1%) and in another, a decrease (lumbar spine − 5.3%, hip − 1.3%). The anecdotal character of this data precludes any conclusions regarding this matter. Although caution should be exerted regarding the interpretation of these features since they only very partially reflect potential harmful effects, VD toxicity does not seem to be associated with long-term adverse outcome in our cohort.

To preclude future dosage mistakes, following the establishment of an accidental overdosing, the pharmacy of each patient was contacted, informed of the error, and reminded about the national guidelines for the preparation of magistral medication. Additionally, given the number of cases, a warning notice regarding the specificities and potential errors associated with magistral preparations of liposoluble vitamins was issued by the Belgian Federal Agency for Medicines and Health Products (FAMHP) and sent to all pharmacists in Belgium. Finally, commercial liposoluble multivitamins have since then been approved for reimbursement by our social security system, probably preventing future accidental intoxications.

Several limitations of our study need to be acknowledged. Firstly, the retrospective, single-center character limits the quality of the data retrieved and its general applicability. Additionally, this retrospective nature implies that even though parameters could be retrieved for most of our population some information is lacking for certain patients (i.e. exact D_3_ dosage in pills, bone mineral density). Secondly, the effect of regional and national specificities regarding medication prescription and magistral preparation habits could influence the frequency of adverse events and hampers generalization of our findings. Nonetheless, this is, to the best of our knowledge, the first study evaluating the prevalence of vitamin D toxicity in a real-life cystic fibrosis population.
